# Amygdalin Blocks Bladder Cancer Cell Growth *In Vitro* by Diminishing Cyclin A and cdk2

**DOI:** 10.1371/journal.pone.0105590

**Published:** 2014-08-19

**Authors:** Jasmina Makarević, Jochen Rutz, Eva Juengel, Silke Kaulfuss, Michael Reiter, Igor Tsaur, Georg Bartsch, Axel Haferkamp, Roman A. Blaheta

**Affiliations:** 1 Department of Urology, Goethe University Hospital, Frankfurt am Main, Germany; 2 Institute of Human Genetics, University Medical Center Göttingen, Göttingen, Germany; Sun Yat-sen University Medical School, China

## Abstract

Amygdalin, a natural compound, has been used by many cancer patients as an alternative approach to treat their illness. However, whether or not this substance truly exerts an anti-tumor effect has never been settled. An in vitro study was initiated to investigate the influence of amygdalin (1.25–10 mg/ml) on the growth of a panel of bladder cancer cell lines (UMUC-3, RT112 and TCCSUP). Tumor growth, proliferation, clonal growth and cell cycle progression were investigated. The cell cycle regulating proteins cdk1, cdk2, cdk4, cyclin A, cyclin B, cyclin D1, p19, p27 as well as the mammalian target of rapamycin (mTOR) related signals phosphoAkt, phosphoRaptor and phosphoRictor were examined. Amygdalin dose-dependently reduced growth and proliferation in all three bladder cancer cell lines, reflected in a significant delay in cell cycle progression and G0/G1 arrest. Molecular evaluation revealed diminished phosphoAkt, phosphoRictor and loss of Cdk and cyclin components. Since the most outstanding effects of amygdalin were observed on the cdk2-cyclin A axis, siRNA knock down studies were carried out, revealing a positive correlation between cdk2/cyclin A expression level and tumor growth. Amygdalin, therefore, may block tumor growth by down-modulating cdk2 and cyclin A. In vivo investigation must follow to assess amygdalin's practical value as an anti-tumor drug.

## Introduction

Bladder carcinoma is the second most common malignancy of the genitourinary tract in western countries, with an incidence of 37.9/100,000 per year for men and 9.6/100,000 per year for women [1]. Approximately 70% of initially diagnosed tumors are superficial and can be treated by transurethral resection, whereby the bladder is preserved. The remaining 30% of tumors become muscle invasive and are associated with a high risk of metastasis [2]. For those patients with locally advanced or metastatic disease, chemotherapy is a treatment option. However, the prognosis of patients with metastases remains poor, with a median survival of 14 months and a 5-year survival rate of 15% [3].

More than 50% of cancer patients in Europe use complementary/alternative medicine (CAM) instead of, or combined with, conventional therapy [4]. Dissatisfaction with conventional treatment and reduction of chemotherapeutic side effects are the most common reasons given for the use of CAM [5], [6]. However, although CAM usage is popular among cancer patients, evidence based benefit from naturally based compounds is lacking. The discrepancy between use of a natural product and knowledge about its anti-tumor properties is notably reflected in the case of amygdalin. Amygdalin (D-mandelonitrile-β-gentiobioside) is a cyanogenic diglucoside present in the pits of many fruits and in numerous plants belonging to the Rosaceae family such as Prunus persica (peach), Prunus armeniaca (apricot) and Prunus amygdalus amara (bitter almond). The term “laetrile” is frequently used as a synonym for amygdalin. However, laetrile is structurally different from the mother compound, amygdalin, and is an acronym (LAEvorotatory and mandeloniTRILE) for a purified, semi-synthetic form of amygdalin. The present investigation employs “amygdalin”.

Amygdalin was one of the most popular, non-conventional, anti-cancer treatments in the 1970s. By 1978, 70,000 US cancer patients had used amygdalin to treat their cancer [7]. Still, evidence based research on amygdalin is sparse and its benefit controversial. Proponents consider amygdalin a natural cancer cure, whereas opponents warn that amygdalin is ineffective and even toxic. Although it has been argued that amygdalin is unsafe, no serious acute toxicity has been encountered. It has also been concluded that amygdalin has no anti-tumor potential, although from 368 cancer patients listed in one review, 12.5% experienced a complete or partial response, 6.8% had stable disease and 22.9% demonstrated symptomatic benefit from amygdalin [8]. To gain insight into how amygdalin might function, an in vitro investigation was initiated to determine its influence on bladder cancer growth and proliferation. Additionally, cell cycle progression and cell cycle regulating proteins were evaluated in amygdalin treated and non-treated cells. siRNA knock down studies were carried out to explore proteins altered by amygdalin, which may have clinical relevance.

The in vitro data presented here point to significant growth and proliferation blocking effects of amygdalin, probably induced by a decrease in the cell cycle regulating proteins cdk2 and cyclin A.

## Materials and Methods

### Cell culture

RT112, UMUC-3 (ATCC/LGC Promochem GmbH, Wesel, Germany) and TCCSUP (DSMZ, Braunschweig, Germany) bladder carcinoma cells were grown and subcultured in RPMI 1640, 10% fetal calf serum (FCS), 20 mM HEPES-buffer, 1% glutamax and 1% penicillin/streptomycin (all: Gibco/Invitrogen; Karlsruhe, Germany). RT112 is an invasive (pathological stage T2) moderately differentiated (grade 2/3) model of human bladder cancer, whereas TCCSUP is a transitional cell carcinoma, grade 4. UMUC-3 represents a high grade 3, invasive bladder cancer. Subcultures from passages 7–24 were used.

### Amygdalin treatment

Amygdalin from apricot kernels (Sigma-Aldrich, Taufkirchen, Germany) was freshly dissolved in cell culture medium and added to tumor cells in concentrations ranging from 1.25–10 mg/ml. Controls remained untreated. In all experiments, treated tumor cell cultures were compared to non-treated cultures. To assess toxic effects of amygdalin, cell viability was determined by trypan blue (Gibco/Invitrogen). For apoptosis evaluation the expression of Annexin V/propidium iodide (PI) was determined using the Annexin V-FITC Apoptosis Detection kit (BD Pharmingen, Heidelberg, Germany). Tumor cells were washed twice with PBS, and then incubated with 5 µl of Annexin V-FITC and 5 µl of PI in the dark for 15 min at RT. Cells were analyzed on a FACScalibur (BD Biosciences, Heidelberg, Germany). The percentage of apoptotic cells (early and late) in each quadrant was calculated using CellQuest software (BD Biosciences).

### Measurement of tumor cell growth and proliferation

Cell growth was assessed using the 3-(4,5-dimethylthiazol-2-yl)-2,5-diphenyltetrazolium bromide (MTT) dye reduction assay (Roche Diagnostics, Penzberg, Germany). Tumor cells (100 µl, 1×10^4^ cells/ml) were seeded onto 96-well tissue culture plates. After 24, 48 and 72 h, MTT (0.5 mg/ml) was added for an additional 4 h. Thereafter, cells were lysed in a buffer containing 10% SDS in 0.01 M HCl. The plates were then incubated overnight at 37°C, 5% CO_2_. Absorbance at 570 nm was measured for each well using a microplate ELISA reader. Each experiment was done in triplicate. After subtracting background absorbance, results were expressed as mean cell number.

Cell proliferation was measured using a BrdU cell proliferation enzyme-linked immunosorbent assay (ELISA) kit (Calbiochem/Merck Biosciences, Darmstadt, Germany). Tumor cells, seeded onto 96-well microtitre plates, were incubated with 20 µl BrdU-labeling solution per well for 8 h, fixed and detected using anti-BrdU mAb according to the manufacturer's instructions. Absorbance was measured at 450 nm.

### Clonogenic assay

Tumor cells, pretreated with amygdalin for 2 weeks, were transferred to 6-well plates at 300 cells per well. Following 10 days incubation, during which the cells were either exposed to amygdalin or not, colonies were fixed and counted. Colonies of at least 50 cells were counted as one.

### Cell cycle analysis

Cell cycle analysis was carried out with subconfluent tumor cells. Tumor cell populations were stained with propidium iodide, using a Cycle TEST PLUS DNA Reagent Kit (BD Pharmingen) and then subjected to flow cytometry with a FACScan flow cytometer (Becton Dickinson). 10,000 events were collected for each sample. Data acquisition was carried out using Cell-Quest software and cell cycle distribution was calculated using the ModFit software (BD Biosciences). The number of gated cells in the G1, G2/M or S-phase is presented as %.

### Western blotting

To investigate proteins involved in cell growth regulation, tumor cell lysates were applied to a 7% polyacrylamide gel and electrophoresed for 90 min at 100 V. The protein was then transferred to nitrocellulose membranes. After blocking with non-fat dry milk for 1h, the membranes were incubated overnight with monoclonal antibodies directed against the cell cycle proteins: Cdk1 (IgG1, clone 1), cdk2 (IgG2a, clone 55), cdk4 (IgG1, clone 97), cyclin A (IgG1, clone 25), cyclin B (IgG1, clone 18), cyclin D1 (IgG1, clone G124–326), p19 (IgG1, clone 52/p19), p27 (IgG1, clone 57; all: BD Pharmingen). The mammalian target of rapamycin (mTOR) pathway was investigated by using the following monoclonal antibodies: anti phospho Rictor (pRictor; IgG, Thr1135, clone D30A3), anti phospho Raptor (pRaptor; IgG, Ser792; both: New England Biolabs, Frankfurt, Germany), anti phospho Akt (pAkt; IgG1, Ser472/Ser473, clone 104A282; BD Pharmingen). Epigenetic modulation was investigated by anti acetylated H3 (aH3; IgG, Lys9, clone C5B11) and anti acetylated H4 (aH4; Lys8, polyclonal, IgG; both: New England Biolabs).

HRP-conjugated goat-anti-mouse IgG (Upstate Biotechnology, Lake Placid, NY, USA; dilution 1∶5.000) served as the secondary antibody. The membranes were briefly incubated with ECL detection reagent (ECLTM, Amersham/GE Healthcare, München, Germany) to visualize proteins and then analyzed by the Fusion FX7 system (Peqlab, Erlangen, Germany). β-actin (1∶1000; Sigma, Taufenkirchen, Germany) served as the internal control.

Gimp 2.8 software was used to perform pixel density analysis of the protein bands. Ratio of intensity of each investigated protein/intensity of β-actin was calculated, and intensity values were then expressed in percentage, related to controls set to 100%.

### Cdk2 and cyclin A knock down

Tumor cells (3×10^5^/6-well) were transfected with small interfering RNA (siRNA) directed against cdk2 (gene ID: 1017, target sequence: AGGTGGTGGCGCTTAAGAAAA) or cyclin A (gene ID: 890, target sequence: GCCAGCTGTCAGGATAATAAA; Qiagen, Hilden, Germany), with an siRNA/transfection reagent (HiPerFect Transfection Reagent; Qiagen) ratio of 1∶6. Non-treated cells and cells treated with 5nM control siRNA (All stars negative control siRNA; Qiagen) served as controls. Subsequently, tumor cell growth was analyzed as indicated above.

### Statistics

All experiments were performed 3–6 times. Statistical significance was evaluated by the non-parametric Wilcoxon–Mann-Whitney-U-test for experiments repeated 6 times. Experiments carried out 3 times were statistically evaluated by the parametric t-test. Differences were considered statistically significant at a p value less than 0.05.

## Results

### Dose-response analysis

**Figure 1:**
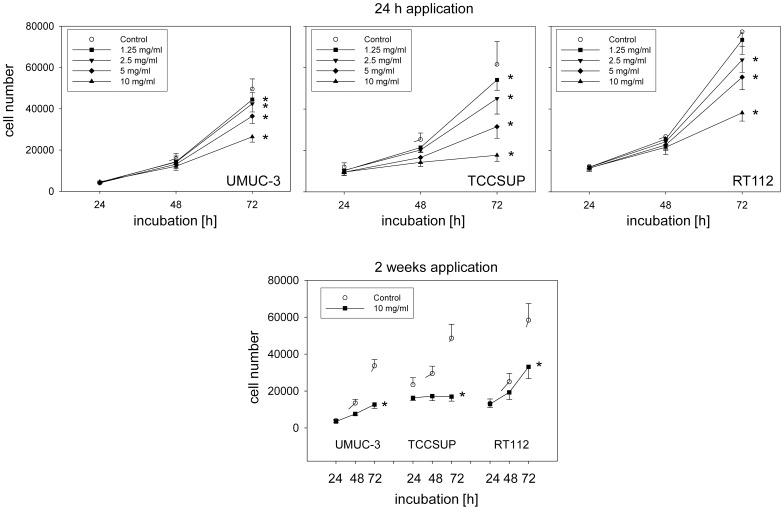
Up: Growth of UMUC-3, TCCSUP and RT112 bladder cancer cells treated with different concentrations of amygdalin after 24 h, 48 h and 72 h. Controls remained untreated. Down: Tumor cell growth after 2 weeks treatment with 10 mg/ml amygdalin. Each experiment was done in triplicate and repeated 5 times. Data from one representative experiment is shown. *indicates significant difference to controls (p = 0.0022).

Exposing the tumor cells to amygdalin (single dose application) led to a concentration-dependent reduction in the tumor cell number, with the most prominent effect apparent in the TCCSUP cell line (fig. 1, 24 h application). No signs of toxicity were shown by the trypan blue exclusion test. Since the strongest cell reduction was seen with 10 mg/ml amygdalin, this concentration was employed for all following investigation. Long-term treatment consisting of 10 mg/ml amygdalin added to tumor cells three times a week over a 2 week period showed that cell growth was diminished to a similar extent as that encountered with the short-term treatment protocol (fig. 1, 2 weeks application).

**Figure 2:**
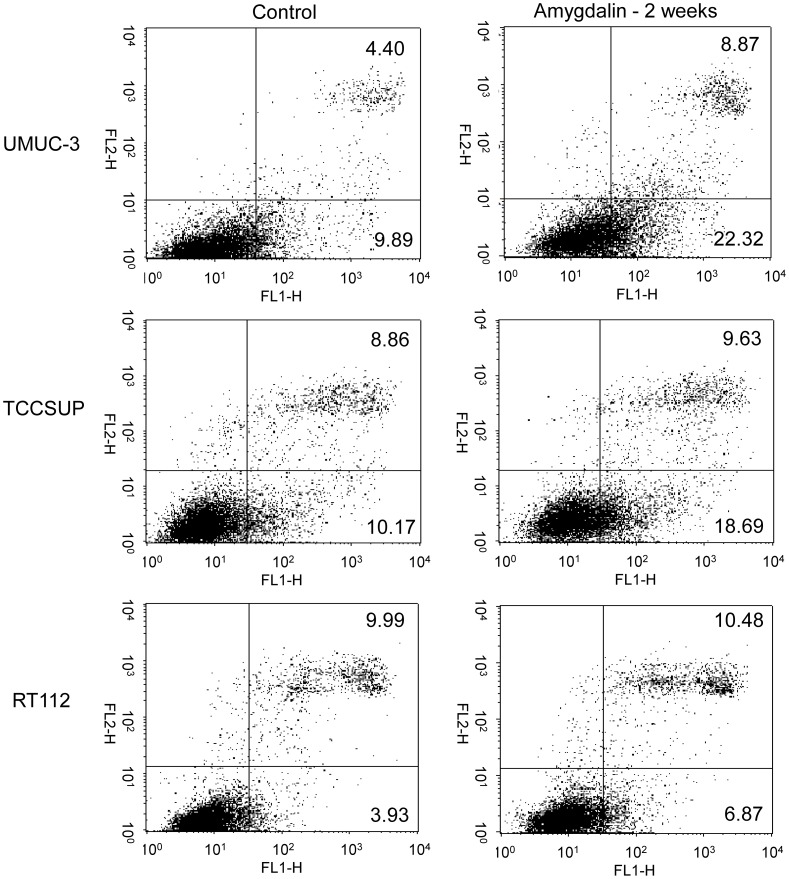
Early and late apoptosis of UMUC-3, TCCSUP and RT112 cells treated with 10 mg/ml amygdalin for 2 weeks. Controls remained untreated. The upper right quadrant shows percentage of cells in late apoptosis, the lower right quadrant percentage of cells in early apoptosis (one representative from 3 tests; SD_intra-assay_ <10%).

### Apoptosis

Short-term application of amygdalin for 24 h did not induce apoptosis (data not shown). However, the percentage of tumor cells undergoing early apoptosis approximately doubled after two weeks amygdalin exposure in all three cell lines: p = 0.0026 (UMUC-3), p = 0.0145 (TCCSUP) and p = 0.0208 (RT112). Additionally, a doubling in late apoptosis occurred in the UMUC-3 cell line (fig. 2; p = 0.0230).

### Tumor cell proliferation and clonal cell growth

**Figure 3:**
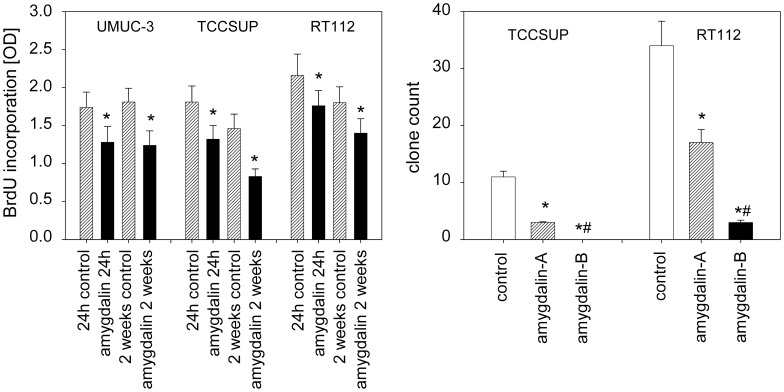
Left: Cell proliferation in UMUC-3, TCCSUP and RT112 cells cultured without (control) and with 10 mg/ml amygdalin for 24 h or 2 weeks (OD = optical density). Right: Clonogenic growth of TCCSUP and RT112 cells (subsequent to 2 week subconfluent culture with 10 mg/ml amygdalin) without (control) and with amygdalin. Amygdalin A = 10 day amygdalin free incubation during clonogenic growth. Amygdalin B = 10 day clonogenic growth with 10 mg/ml amygdalin. Experiments were done in triplicate and repeated 5 times. *indicates significant difference to controls (p = 0.0022). ^#^indicates significant difference to amygdalin A (p = 0.0022).

Proliferation in all three cell lines was significantly decreased, whether amygdalin was applied for 24 h or 2 weeks (figure 3, left). Following 2 weeks amygdalin exposure to subconfluent cells, clonal growth was then evaluated, whereby amygdalin was either added for a subsequent period of 10 days (“amygdalin B”) or not (“amygdalin A”). The number of RT112 clones was considerably diminished by 10 day amygdalin exposure during clonal formation, and no TCCSUP clones were apparent (fig. 3, right). When amygdalin was not applied during clonal formation, the number of RT112 and TCCSUP clones was also reduced, though not as strongly as was seen in the “amygdalin B” regimen (fig. 3, right). The UMUC-3 cell line did not form colonies and was, therefore, not evaluated.

### Cell cycle progression

**Figure 4:**
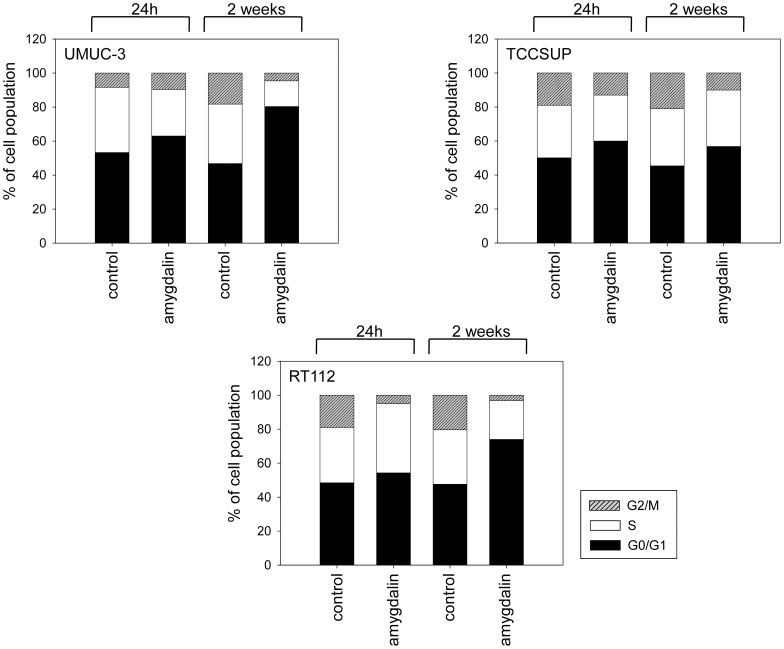
Cell cycle analysis of UMUC-3, TCCSUP and RT112 subconfluent cultures pretreated with amygdalin for 24 h or 2 weeks (controls remained untreated). The cell population is expressed as percentage of the total cells analyzed. One representative experiment of three is shown.

Amygdalin modulated cell cycle progression, depending on the tumor cell line (fig. 4). Short-term amygdalin application (24 h) to UMUC-3 increased the number of G0/G1 cells (p = 0.0169) and reduced the number of S-phase cells (p = 0.0138). In TCCSUP cells short-term treatment increased the number of G0/G1-phase cells (p = 0.0121), but reduced the number of cells in the G2/M-phase (p = 0.0406). In RT112 cells short-term amygdalin exposure caused G2/M-phase reduction (p = 0.0039) and an S-phase increase (p = 0.0040). Two weeks amygdalin exposure lowered the G2/M-phase (p_UMUC-3_ = 0.0036; p_TCCSUP_ = 0.0080; p_RT112_ = 0.0015) and increased the G0/G1-phase in all tumor cell lines (p_UMUC-3_ = 0.0019, p_TCCSUP_ = 0.0143, p_RT112_ = 0.0018). The number of S-phase cells was also significantly diminished after two weeks treatment in UMUC-3 (p = 0.0093) and RT112 cells (p = 0.0032), compared to controls.

### Cell cycle regulating protein expression

**Figure 5:**
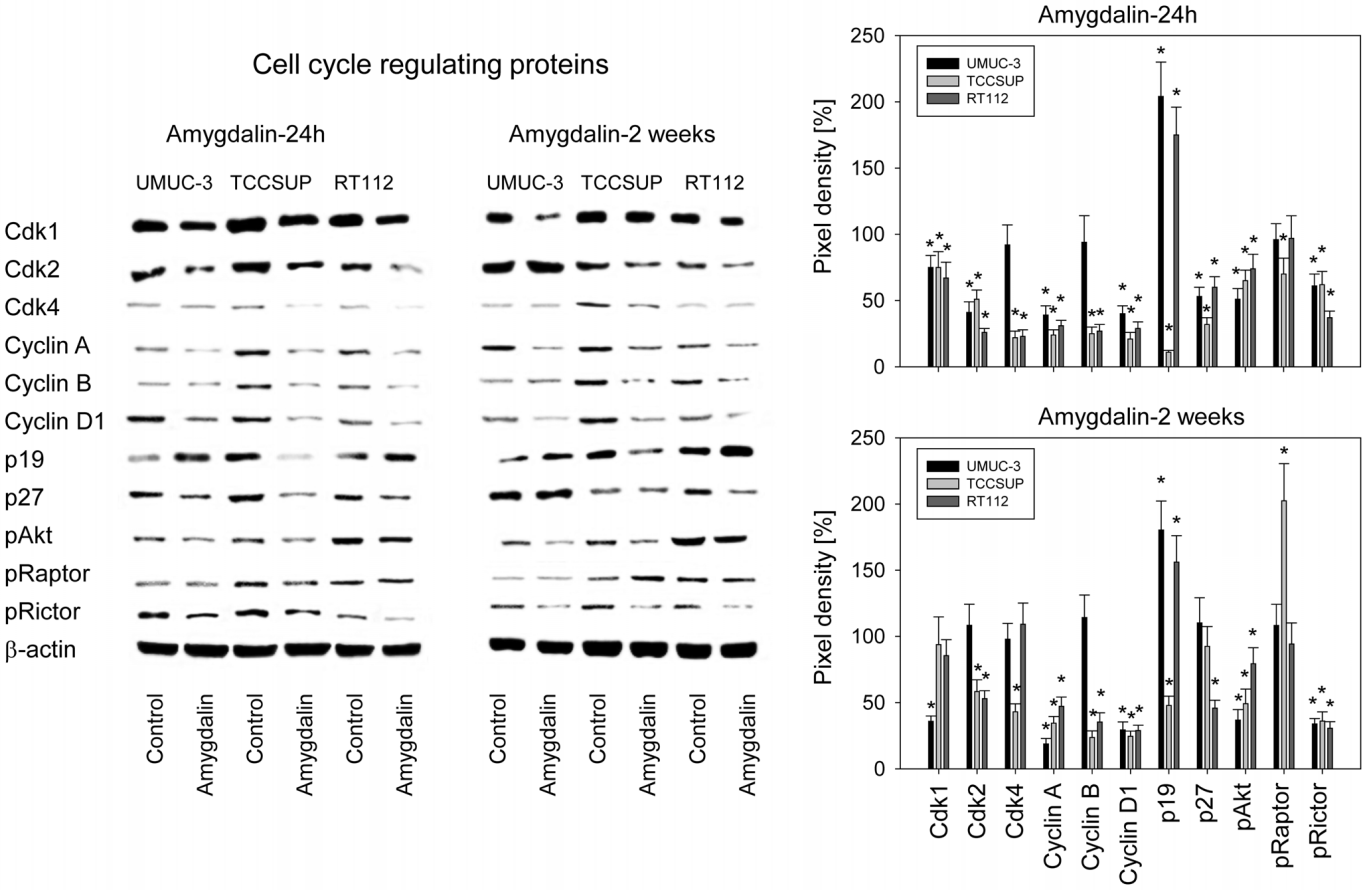
Western blot of cell cycle and mTOR related proteins from lysates of UMUC-3, TCCSUP and RT112 cell lines. Tumor cells were pretreated with amygdalin for 24 h or 2 weeks (controls remained untreated). β-actin served as the internal control. One representative from three separate experiments is shown. The right panel of figure 5 shows pixel density values given in percentage related to controls not treated with amygdalin. *indicates significant difference to the control.

Since amygdalin influenced cell growth and cell cycle progression, modifications of the cell cycle controlling proteins could be expected. The expression of cdk1 and cdk2 (all cell lines) and cdk4 (TCCSUP, RT112) was diminished 24 h after amygdalin application (fig. 5). P-values were as follows for cdk1 (p_UMUC-3_ = 0.0029, p_TCCSUP_ = 0.0052, p_RT112_ = 0.0007), for cdk2 (p_UMUC-3_ = 0.0001, p_TCCSUP_ = 0.0006, p_RT112_ = 0.0001), for cdk4 (p_TCCSUP_ = 0.0006, p_RT112_ = 0.0001), for cyclin A (p_UMUC-3_ = 0.0001, p_TCCSUP_ = 0.0001, p_RT112_ = 0.0002), for cyclin B (p_TCCSUP_ = 0.001, p_RT112_ = 0.0001) and for cyclin D1 (p_UMUC-3_ = 0.0001, p_TCCSUP_ = 0.0001, p_RT112_ = 0.0001). p19 was enhanced in UMUC-3 (p = 0.0013) and RT112 (p = 0.0012), but reduced in TCCSUP (p = 0.0001), whereas p27 was reduced in all cell lines (p_UMUC-3_ = 0.0013, p_TCCSUP_ = 0.0001, p_RT112_ = 0.0001). pAkt as well as pRictor (but not pRaptor), from the mTOR pathway, were deactivated in all three cell lines. P-values were as follows for pAkt (p_UMUC-3_ = 0.0002, p_TCCSUP_ = 0.0004, p_RT112_ = 0.0082) and for pRictor (p_UMUC-3_ = 0.0015, p_TCCSUP_ = 0.0017, p_RT112_ = 0.0001). pRaptor was diminished only in TCCSUP (p = 0.0024).

Slight differences were apparent after 2 weeks as opposed to the 24 hour amygdalin exposure. Cdk1 but not cdk2 was down-regulated in UMUC-3 (p = 0.0001). The 24 h diminishing influence of amygdalin on p27 expression in UMUC-3 and TCCSUP cells was lost after 2 weeks, and pRaptor increased after 2 weeks in TCCSUP cells (p = 0.0019), in contrast to the 24 h diminishing effect (fig. 5).

### Cdk2/cyclin A knockdown

**Figure 6:**
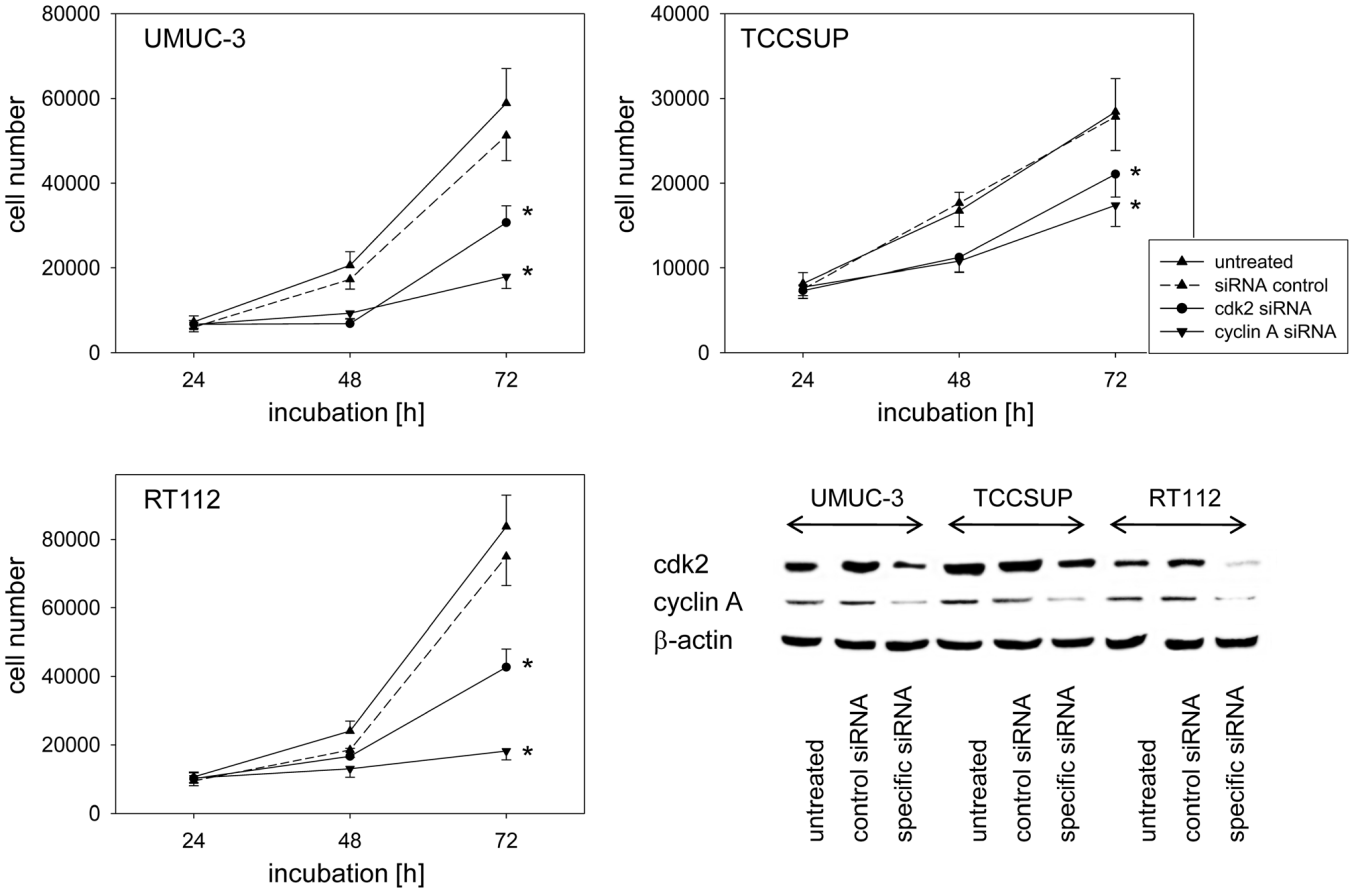
Influence of cdk2 or cyclin A knock down on tumor cell growth. UMUC-3, TCCSUP and RT112 cells were transfected with cdk2 or cyclin A siRNA and knock down was controlled by western blot (lower right). One representative from 6 experiments is shown. *indicates significant difference to controls (p = 0.0022).

Since amygdalin strongly modified cdk2 and cyclin A in all tumor cell lines and these proteins regulate entry into the mitotic cycle, the role of these proteins in tumor growth was evaluated by siRNA knock-down. Incubation of UMUC-3, RT112 and TCCSUP cells with siRNA against cdk2 or cyclin A distinctly decreased the protein content (fig. 6, lower right) and was accompanied by significant growth blockade in all three cell lines (fig. 6).

## Discussion

Evidence presented here shows that amygdalin suppresses growth and proliferation in three bladder cancer cell lines. The number of tumor cells undergoing early apoptosis slightly increased after long-term amygdalin exposure, though not after short-term exposure and this inhibition of proliferative activity was not caused by a toxic effect of amygdalin. Other investigators have also shown signs of apoptotosis induced by amygdalin. Rapid activation of caspase-3 along with down-regulation of Bcl-2 and up-regulation of Bax in the presence of 0.1 mg/ml amygdalin has been demonstrated in DU145 and LNCaP prostate cancer cells [9]. Apoptotic cell death has also been evoked by amygdalin in the cervical cancer cell line HeLa [10] and in human promyelocytic leukemia (HL-60) cells [11]. Altogether these reports indicate that amygdalin may account for apoptotic events in diverse cancer cells, including those from bladder, and contribute to reduced tumor growth.

Amygdalin strongly altered cell cycle progression in all three bladder cancer cell lines. During short-term treatment, UMUC-3 and TCCSUP accumulated in G0/G1, whereas RT112 was arrested in the S-phase. Mitosis is therefore influenced in a different manner in different cell lines by amygdalin. Another difference was that p19 increased in RT112 but decreased in TCCSUP. Rictor was deactivated and cdk4 was suppressed in RT112 but not in UMUC-3. Cdk4 inhibition and p19 increase has recently been demonstrated to drive tumor cells into the S-phase [12]. Since both Cdk4 inhibition and p19, in combination, only occurred in the RT112 cells, this could explain why RT112 accumulated in the S-phase, rather than in the G0/G1 phase. Indeed, cdk4 was not reduced after 2 weeks amygdalin application, and RT112 cells were then arrested at G0/G1. Nevertheless, S-phase entry is not exclusively controlled by cdk4 and p19. Rather, a cohort of cell cycle proteins is involved in driving cell division forward. Amygdalin seems, therefore, to interfere in a cell line dependent manner with several checkpoint molecules, disturbing the fine-tuned mitotic machinery. Growth blockade is the final result.

The relevance of p19 and p27 in bladder cancer progression has not been fully elucidated. Both proteins were strongly modified after amygdalin application in this investigation, p27 being down-regulated in all cell lines, p19 being up-regulated in UMUC-3 and RT112 but diminished in TCCSUP. Semi-quantitative RT-PCR on a series of 32 bladder cancer specimens paired with adjacent normal tissues has not revealed any significant differences of p19 and p27 expression [13]. On the other hand, prospective evaluation of high-grade bladder cancer patients treated with radical cystectomy demonstrated more p27 positive than negative tumors in patients with disease recurrence [14]. Microarray analysis has shown a positive correlation between p27 expression and advanced bladder cancer [15]. In contrast, another study has reported an unfavorable effect of p27 loss associated with bladder carcinoma [16]. In vitro experiments have pointed to an indeterminate role of p27 in as much as suppression of this protein elevated growth but simultaneously reduced invasion [17]–[19]. Based on this, it is not possible to finally assess whether p27 reduction after amygdalin application is linked to proliferation, to invasion or to both. To gain insight into the invasion process, a new investigation has been initiated concentrating on the action of amygdalin on cancer cell spreading.

Amygdalin reduced phosphorylation of Akt and of the mTOR subunit, rictor. Akt-mTOR signaling plays a major role in bladder carcinogenesis, with Akt activation occurring over the entire spectrum of bladder urothelial carcinomas [20]. The Cancer Genome Atlas project has identified the AKT/mTOR pathway as a critical therapeutic target in bladder cancer [21]. The specific action of amygdalin on the mTOR complex rictor is of interest, since the approved mTOR-inhibitors, everolimus and temsirolimus, target the mTOR complex raptor, whereas rictor is considered insensitive to both drugs [22]. Rictor has been shown to be an important determinant in bladder cancer migration and invasion [23]. Thus, amygdalin might not only be an innovative tool to neutralize metastatic dissemination but also to complement mTOR-inhibitor based regimens.

Cdks, along with cyclins, are key molecules responsible for cell cycle progression and cell division, whereby particular cdks are affiliated with particular cyclins. Consequently, aberrant cancer cell growth has been attributed to modulation in the cdk-cyclin expression level. The strongest protein alterations after 24 h amygdalin incubation were observed for cdk2 and cyclin A, and may represent major targets of amygdalin. Though no investigators to date have advanced this hypothesis, amygdalin has been shown to alter genes involved in cell cycle regulation of human colon cancer cells [24]. The cdk2-cyclin A axis promotes G1/S phase transition, which might explain why cdk2/cyclin A down-modulation by amygdalin was accompanied by a G0/G1 phase arrest in UMUC-3 and TCCSUP cells. In vitro investigation of soft tissue sarcoma cells has shown that cdk2 decrease combined with p27 loss affects the cellular invasion program [25]. Since cdk2 reduction was paralleled by a p27 decrease in the bladder cancer model, it seems likely that amygdalin not only acts on tumor growth but could also influence metastatic spread.

Indeed, although cdk2-cyclin A are altered in many solid tumors, information about cdk2-cyclin A in bladder cancer is sparse. Protein and mRNA analyses of bladder carcinoma tissues have indicated that cdk2 is closely associated with bladder tumor development and progress [26]. Cdk2's binding partner, Cyclin A, has been correlated with tumor grade and poor disease-specific survival, evidenced by immunohistochemistry and cDNA microarrays [27]. In the current experiments, knocking down cdk2 or cyclin A led to a substantial reduction of the tumor cell number, indicating the clinical relevance of both proteins for bladder cancer. Since cdks and cyclins are enhanced when drug resistance develops [28], [29], counteracting this process might be a potent strategy to prevent or overcome resistance [29]. During the last years, several novel therapeutic strategies have been designed to target cdks. Currently, they are in various stages of clinical development, as both single agents and in combination [30]. Amygdalin could be a “natural” alternative, whereby severe side-effects associated with conventional cdk-inhibitors [31] might be avoided.

Slight differences between short-term and long-term amygdalin application were observed, particularly in the UMUC-3 cell line. Cdk2 was no longer diminished after 2 weeks, but cdk1 was more strongly decreased than after 24 h amygdalin application. The reason for this switch over after long-term application is not clear. Cdk2 accumulates at the G1/S phase boundary, whereas cdk1 drives cells into mitosis [32]. The diverse mechanisms of cdk1 and cdk2 have been reflected by the cell cycle assay demonstrating additional arrest of UMUC-3 in G2/M after 2 weeks amygdalin exposure. Since UMUC-3 growth and proliferation was similarly altered in the 24 h and 2 weeks amygdalin application, cdk2 may have become insensitive to amygdalin suppression, but this was compensated for by a suppression of cdk1. In fact, cdk1 has been reported to compensate for cdk2 by redirecting cdk1 into the cyclin A pathway, which is normally restricted to cdk2 [33].

This is the first investigation providing information about amygdalin's influence on bladder cancer cell lines in vitro. Such in vitro studies can do no more than put forward a prediction of amygdalin's efficacy in patients. Clinical trials with amygdalin have not been well documented and randomized controlled studies have never been carried out. A retrospective analysis of 67 tumor patients who had taken amygdalin reported two complete and 4 partial responses [34]. A phase II trial was conducted in 1982, whereby patients also received vitamins and pancreatic enzymes. Therapy was stopped when blood cyanide levels increased and it was concluded that Laetrile was not an effective cancer treatment [35]. Other reports suggest amygdalin to be of clear benefit in cancer patients [8]. However, the term “benefit” was not clearly specified. Ambivalence has also been reflected in case reports, where amygdalin was ineffective in five and effective in four cases [8].

It is still not clear whether amygdalin might also act on normal, physiologically intact epithelial cells. Since primary non-immortalized epithelial cells do not show mitotic activity or rapidly lose mitotic activity in vitro, these cells cannot be subjected to the MTT growth assay. However, amygdalin has recently been demonstrated to block the growth of endothelial cells [36]. Whether this phenomenon can be transferred to other cells remains open. Nevertheless, blockage of endothelial cell growth by amygdalin indicates that amygdalin may suppress tumor induced angiogenesis.

The risk of developing cyanide poisoning has also not been resolved. In this respect the route of administration (oral or intravenous), quantity and quality will surely influence the risk-benefit ratio. Since clinical reports of amygdalin treatment are more than 30 years old [8], and during this time the understanding of molecular cancer mechanisms has greatly progressed, it would be worthwhile to test the in vitro suppositions presented here in an vivo model. Well-designed, controlled clinical trials to critically test amygdalin should then be considered.

Overall, amygdalin has been shown to block the growth of bladder cancer cells in vitro. Suppression of cdk2 and cyclin A might be one relevant mechanism defining how amygdalin may arrest or diminish tumor proliferation.
